# Correction: Parental migration and psychological well-being of left-behind adolescents in Western Nepal

**DOI:** 10.1371/journal.pone.0250836

**Published:** 2021-04-22

**Authors:** Madhu Kharel, Akira Shibanuma, Junko Kiriya, Ken Ing Cherng Ong, Masamine Jimba

The second author’s name is incorrect. The correct name is: Akira Shibanuma. The correct citation is: Kharel M, Shibanuma A, Kiriya J, Ong KIC, Jimba M (2021) Parental migration and psychological well-being of left-behind adolescents in Western Nepal. PLoS ONE 16(1): e0245873. https://doi.org/10.1371/journal.pone.0245873
