# Supplementary material for: Parental migration and psychological well-being of left-behind adolescents in Western Nepal
Source: PLoS One. 2021 Jan 28;16(1):e0245873. doi: 10.1371/journal.pone.0245873 (PMC7842897; doi:10.1371/journal.pone.0245873)
Supplement: S2 File — (DOCX) [file pone.0245873.s002.docx]

**Table 1 a. General characteristics of adolescents by parental migration status (n = 626).**

| Variables | | Total | | Non-migrant parents | | One parent migrant | | Both parents migrant | |
| --- | --- | --- | --- | --- | --- | --- | --- | --- | --- |
|  |  | n | %* | n | %* | n | %* | n | %* |
| Age (years) Mean (SD) | | 14.3 (1.2) | | 14.3 (1.2) | | 14.2 (1.2) | | 14.3 (1.3) | |
| Age group | | | | | | | | | |
|  | Early adolescence (10-14 years) | 354 | 56.6 | 215 | 60.7 | 98 | 27.7 | 41 | 11.6 |
|  | Late adolescence (15-19 years) | 272 | 43.5 | 168 | 61.8 | 68 | 25.0 | 36 | 13.2 |
| Gender | | | | | | | | | |
|  | Male | 300 | 47.9 | 182 | 60.7 | 78 | 26.0 | 40 | 13.3 |
|  | Female | 326 | 52.1 | 201 | 61.7 | 88 | 27.0 | 37 | 11.4 |
| Type of school | | | | | | | | | |
|  | Public | 457 | 73.0 | 306 | 67.0 | 122 | 26.7 | 29 | 6.4 |
|  | Private | 169 | 27.0 | 77 | 45.6 | 44 | 26.0 | 48 | 28.4 |
| Religion | | | | | | | | | |
|  | Hindu | 607 | 97.0 | 367 | 60.5 | 164 | 27.0 | 76 | 12.5 |
|  | Buddhist | 11 | 1.8 | 9 | 81.8 | 2 | 18.2 | 0 | 0.0 |
|  | Christian | 6 | 1.0 | 5 | 83.3 | 0 | 0.0 | 1 | 16.7 |
|  | Muslim | 1 | 0.2 | 1 | 100.0 | 0 | 0.0 | 0 | 0.0 |
|  | Other | 1 | 0.2 | 1 | 100.0 | 0 | 0.0 | 0 | 0.0 |
| Ethnicity | | | | | | | | | |
|  | Brahmin | 106 | 16.9 | 76 | 71.7 | 15 | 14.2 | 15 | 14.2 |
|  | Chhetri | 234 | 37.4 | 108 | 46.2 | 73 | 31.2 | 53 | 22.7 |
|  | Janajati | 173 | 27.6 | 124 | 71.7 | 44 | 25.4 | 5 | 2.9 |
|  | Dalit | 112 | 17.9 | 74 | 66.1 | 34 | 30.4 | 4 | 3.6 |
|  | Muslim | 1 | 0.2 | 1 | 100.0 | 0 | 0.0 | 0 | 0.0 |
| Father's education | | | | | | | | | |
|  | Did not complete primary level | 113 | 18.1 | 84 | 74.3 | 23 | 20.4 | 6 | 5.3 |
|  | Completed primary or secondary level | 424 | 67.7 | 238 | 56.1 | 131 | 30.9 | 55 | 13.0 |
|  | Completed higher than secondary level | 89 | 14.2 | 61 | 68.5 | 12 | 13.5 | 16 | 18.0 |
| Mother's education | | | | | | | | | |
|  | Did not complete primary level | 178 | 28.4 | 139 | 78.1 | 32 | 18.0 | 7 | 4.0 |
|  | Completed primary or secondary level | 385 | 61.5 | 209 | 54.3 | 117 | 30.4 | 59 | 15.3 |
|  | Completed higher than secondary level | 63 | 10.1 | 35 | 55.6 | 17 | 27.0 | 11 | 17.5 |
| Parental migration status | | | | | | | | | |
|  |  | 662 | 100.0 | 383 | 61.2 | 166 | 26.5 | 77 | 12.3 |
| Relationship with primary caretaker | | | | | | | | | |
|  | Very good | 584 | 93.3 | 336 | 62.7 | 155 | 26.5 | 63 | 10.8 |
|  | Satisfactory | 39 | 6.2 | 16 | 41.0 | 10 | 25.6 | 13 | 33.3 |
|  | Poor | 1 | 0.2 | 0 | 0.0 | 0 | 0.0 | 1 | 100.0 |
|  | Very poor | 2 | 0.3 | 1 | 50.0 | 1 | 50.0 | 0 | 0.0 |

SD, standard deviation

* Total might not add up to 100 due to rounding off.
