# Supplementary material for: Parental migration and psychological well-being of left-behind adolescents in Western Nepal
Source: PLoS One. 2021 Jan 28;16(1):e0245873. doi: 10.1371/journal.pone.0245873 (PMC7842897; doi:10.1371/journal.pone.0245873)
Supplement: S3 File — (DOCX) [file pone.0245873.s003.docx]

**Coding of study variables**

| **Variable** | **Code in the data set** | **Categories** |
| --- | --- | --- |
| Age | *age* | Continuous |
| Age group | *agegroup* | 1. Early adolescence 2. Late adolescence |
| Gender | *sex* | 1. Male 2. Female 3. . (Missing) |
| Type of school | *school_type* | 1. Public 2. Private |
| Religion | *religionn* | 1. Hindu 2. Buddhist and other 3. . (Missing) |
| Ethnicity | *ethnicityn* | 1. Brahmin 2. Chhetri and other 3. . (Missing) |
| Father's Education | *fat_edu* | 1. Did not complete primary level 2. Completed primary or secondary level 3. Completed higher than secondary level |
| Mother's Education | *mot_edu* | 1. Did not complete primary level 2. Completed primary or secondary level 3. Completed higher than secondary level 4. . (Missing) |
| Parental migration status | *parmigstaf* | 1. None of the parents living abroad 2. One of the parents living abroad 3. Both parents living abroad |
| Relationship with the primary caretaker | *pricaretaker_relationn* | 1. Very good 2. Not very good 3. . (Missing) |
| Wealth quintile | *wealth5* | 1. 1 2. 2 3. 3 4. 4 5. 5 |
