# Supplementary material for: Parental migration and psychological well-being of left-behind adolescents in Western Nepal
Source: PLoS One. 2021 Jan 28;16(1):e0245873. doi: 10.1371/journal.pone.0245873 (PMC7842897; doi:10.1371/journal.pone.0245873)
Supplement: Attachment — Submitted filename: Response to Reviewers.docx [file pone.0245873.s004.docx]

**Response to Reviewer #1:**

Results, 1st paragraph: It may be clearer if you state that all children present on the days of the surveys completed the questionnaire. However, for analysis, those with divorced/deceased parents were excluded. At the moment it is slightly unclear from the way you have described it.

We have revised the section accordingly. (Page 9, Line: 168-170)

"All the children present on the days of the survey completed the questionnaire. However, for analysis, those with divorced, deceased and internal migrant parents were excluded."

Results, Table 1. I would find it helpful to see these demographic characteristics according to parental migrant status. Would it be possible to add to this table (or in an additional table) the distribution of these characteristics by (a) whole sample; (b) one-parent migrant; (c) both parent migrants; (d) non-migrant?

We agree with the reviewer's idea. We have added a supplementary table. (SF 2).

Results, Table 3. It is interesting that you chose 1-migrant-parent as the reference group. Please can you explain rationale for this? I would imagine it would make more sense to use non-migrant parent group as the reference, because it is the largest group and also it is most relevant to the overall research question - i.e. do children with migrant parents have worse outcomes than those with no migrant parents. It is slightly more difficult to interpret when the reference category is 1-parent, and you are comparing those to either no-migrant or 2-parents.

We appreciate reviewer's suggestion. However, in our study, 97.6% of one-migrant parents (162 of 166) were fathers. In low income settings, as in this study, fathers working abroad would be a good source of financial security for their children and families. At the same time, mothers staying together with the children would be able to provide the love and care they need. So, this group of adolescents would have better mental well-being compared to the other two groups. Therefore, we chose this group as the reference group.

Results, Table 3. I also cannot understand why in the simple linear regression, the order of scores changes. In Table 2, 1-parent group has the lowest SDQ scores, followed by non-migrant, and finally 2-parents. In Table 3, this has changed to lowest risk in non-migrant, followed by 1-parent, followed by 2-parents. What has changed, given that in the simple regression model you are (presumably) not controlling for any other factors yet?

Results in Table 3 are consistent with those in Table 2. In Table 3, the coefficient (B) for non-migrant parents group i.e. 0.68 indicates that the mean total difficulty score in non-migrant parents group is 0.68 higher than in the one-parent migrant group (reference group). The coefficient (B) for both-parents migrant group i.e. 1.74 indicates that the mean total difficulty score in both-parents migrant group is 1.74 higher than in the one-parent migrant group (reference group).

**Response to Reviewer #2:**

1. On page 4, line7, your paper argues that most studies focus on the left behind children in China in an internal migration context. However, there are now more studies on the psychological well-being of left behind children in Africa, Latin America and other parts of Asia (the Philippines, Indonesia). It is important that you acknowledge and get insights from these quantitative studies, which will help you in improving the choice of variables for your analysis and improving the key conclusions you can draw from this analysis.

Thank you for your suggestion. We went through those papers during the literature review and got useful insights from them. We have added more information in the relevant section of the text. (Page: 4 and 5, Line: 70-75)

"Apart from the studies in China, studies have been conducted in some parts of Asia and Africa to see the impact of parental international migration on the mental health of left-behind children and adolescents [8, 9, 13]. However, these studies have produced mixed results, with some reporting negative effects of parental migration [8, 9, 13] while others reporting no difference in the psychological well-being of adolescents [8, 13]. Moreover, only a few studies have focused on adolescents."

2. In the Methods section,
   a. the use of psychological data on the African adolescents is neither properly explained nor very well justified. It is not particularly clear for what purpose you use this data and what insight it can bring to the study, as well on what basis Nepalese and African Adolescents can be compared. Note: it is very important that you mention the exact country in Africa that this data represents as Africa is a continent covering many countries. If you are comparing continental average data, it is important to explicit about that.

Thank you for your concern. We used the data on psychological well-being of adolescents from a study among adolescents in Angola. We could not find studies from Nepal or other Asian countries which had relevant information on adolescents' psychological well-being to calculate the sample size for the study. We believe adolescents from low- and middle-income countries (Nepal and Angola both being low- and middle-income countries) share similar problems to some extent. We have revised the text in the manuscript to make it clearer. (Page: 6, Line: 95-101)

"Sample size was calculated using OpenEpi software based on the findings from a similar study in Angola in Africa (a lower-middle income country as Nepal) as studies from Nepal or other Asian countries were not found which had relevant information on adolescents' psychological well-being to calculate the sample size for the study. In Angola, the adolescents living with both parents had mean psychological well-being score of 13.0 and standard deviation (SD) of 5.6. These figures for the adolescents with both parents away were 15.1 and 5.9, respectively [13]."

b. The methods section should be explicit about what kind of information were collected in the survey.

We have clearly explained in the "Measures" part of the "Methods" section about what information were collected in the survey - Parental migration status, relationship with primary caretaker, psychological well-being of adolescents and sociodemographic characters (Page: 7 and 8, Line: 119-143).

To make it clearer, we have added a summary paragraph at the beginning of "Measures" part of the "Methods" section. (Page: 7, Line: 116-118)

"In this study, the exposure variable was parental migration status and the outcome variable was psychological well-being of adolescents. Information on adolescents' perceived relationship with their primary caretaker and other socio-demographic variables were also collected."

c. It is important to add a supplementary table on the coding of the socioeconomic and demographic variables.

We have added a supplementary table on the coding of study variables. (SF 3)

d. Because you are focusing on the left behind children, it is crucial that you account for the characteristics of the caregiver: is the caregiver employed, other than relationship with the caregiver. It is also important to control for the economic activities of these children (see #3 comment for more explanation). Please also consider variables such as household size if it is available.

Thank you for your suggestion. In this study, we did not collect further information about the primary caregiver as we thought that the relationship with the primary caregiver is the most important factor for adolescents' mental well-being. We could consider this in our future studies. We have added this limitation to the main text. (Page: 19, Line: 302-304)

"Fourth, the characteristics of primary caretakers, other than their relationship with the adolescents, were not captured in this study."

We also do not have information on the household size. With regards to adolescents' engagement in economic activities, adolescents in Nepal usually do not engage in income-generating activities on their own such as doing part-time jobs. They mostly support their families with household work.

e. In the analyses strategy, it is important to conduct further analyses to check the robustness of your results. Contrary to findings for Ghanaian and Angolan left behind children, your study finds that adolescents living with both their parents have lower psychological well-being. I suggest you conduct separate analyses for girls and boys to see whether this result persists. You can also conduct separate analyses for early adolescent and late adolescent groups as the effect of parental migration could differ between these groups.

We agree to the reviewer's opinion that a further analysis would be helpful. However, if we conduct a sub-group analysis, the study will not have sufficient power to detect the difference due to small sample size. If the sample size was bigger, we could have done it. However, we have controlled for adolescents' gender and age in the multiple linear regression analysis. In addition, we have added this limitation in the main text. (Page: 19 and 20, Line: 304-306)

"Next, sub-group analysis could not be performed for adolescents' gender and age group as the study will not have enough power to detect the difference due to small sample size."

3. To explain the results you find, it might be wise to look into childhood and youth studies, which study these groups in non-migratory contexts. It is also worthwhile to include some background information about Nepal’s socioeconomic and adolescent labor conditions to understand the emigration context better. That could for instance help to nuance why adolescents living with both parents could be in a worse situation. It could be that they may themselves need to work to support their families’ livelihood.

Thank you for raising an important point. We have added this information in the main text. (Page: 17, Line: 250-257)

"Generally, the school-going adolescents in Nepal are not engaged in direct income-generating activities. However, most of them support their families with household work. Agriculture is the primary occupation of majority of the families in the study area. Families without foreign income will have to work more in the field to sustain their livelihoods. Accordingly, adolescents in such families have to work extra hours to support their families. In some families with poor economic status, the older adolescents take paid work in their free time or during holidays to support their families economically. This extra burden on adolescents can affect their psychological well-being."
