# Supplementary material for: Parental migration and psychological well-being of left-behind adolescents in Western Nepal
Source: PLoS One. 2021 Jan 28;16(1):e0245873. doi: 10.1371/journal.pone.0245873 (PMC7842897; doi:10.1371/journal.pone.0245873)
Supplement: Attachment — Submitted filename: Response to Reviewers.docx [file pone.0245873.s005.docx]

**Response to Reviewer #2:**

1. It is not clear why you call Table 3 a simple linear regression analyses and Table 4 a multiple linear regression analyses. A simple linear regression is when you only have one variable in your analyses. By this logic, Table 3 is rather a multiple linear regression table. It is also not clear why and how Table 3 and Table 4 differ. I can see that the regression results on the parental migration status variable are not the same in the two tables even though the control variables used are the same. Table 4 shows that children living with both parents in Nepal have a higher SDQ compared to those with one of their parents abroad whereas Table 3 shows that an increased SDQ for children with both parents abroad. What is your explanation for these two opposite findings?

We call Table 3 a simple linear regression analyses because in Table 3, we present the results of bivariate analyses between each independent variable and the dependent variable (i.e. psychological well-being), without controlling for other independent variables. We call table 4 a multiple linear regression analyses because we have controlled for other independent variables while looking at the association between each independent variable and the dependent variable. [UCLA, 2020]

Table 4 is different from Table 3 in that the Table 3 presents the results of a crude association between each independent variable and the dependent variable while Table 4 shows the results after controlling for potential confounders.

Results from the simple linear regression analyses (Table 3) show that adolescents living with both parents tend to have a higher SDQ compared to those with one of their parents living abroad. However, when we controlled for potential confounders (Table 4), adolescents living with both parents tend to have a higher SDQ compared to those with one of their parents abroad. Therefore, the different results are due to confounding.

We have removed Table 3 and presented only Table 4 (as Table 3 in the revised manuscript).

*Reference:*

UCLA Institute for Digital Research and Education Statistical Consulting. Regression with STATA Chapter 1 - Simple and multiple regression [Internet]. California: UCLA Institute for Digital Research and Education; [cited: 2020 Dec 19]. Available from: https://stats.idre.ucla.edu/stata/webbooks/reg/chapter1/regressionwith-statachapter-1-simple-and-multiple-regression/

2. It might be worth discussing whether adolescents girls' and boys' SDQs respond differently due to their parent(s)' migration. To do that my suggestion is to run separate regressions for the girls' and boys' sub-samples. Alternatively, you may choose to see the interaction effect of gender and the parental migration status variable. This could give your analyses more nuance.

Thank you for your suggestion. We ran multiple regression analysis including the interaction term for gender and parental migration status. However, the interaction term was not statistically significant. Therefore, we did not include it in the final model.

3. Your conclusion focuses a lot on general discussions on other control variables. It is best if your conclusion dedicates more time contemplating on the result on the parental migration status variable by using some contextual information to explain the result.

Thank you for your suggestion. In the revised manuscript, we have discussed more about the parental migration status variable, and shortened the discussion on other control variables.

Please refer to page: 16-18, line: 238-288.

4. Suggestion: if you make the conclusion section succinct, then you can have more space to include some background information about parental migration in Nepal either in the introduction section or as a separate section and come back to this in the conclusion section.

Thank you for your suggestion. We have separated the conclusion section to make it clear. We have also added background information about parental migration in Nepal in the introduction section.

Conclusion Section:

(Page 19, Line: 314-318)

***Conclusion***

*In rural districts of Nepal, where international migration is common, adolescents living with the parents were more likely to have a poorer psychological well-being compared to those with one of the parents living abroad. Future studies could explore adolescents' adaptation mechanism for the absence of parents due to international migration.*

Introduction Section:

(Page: 4, Line: 56-58)

*"Nepal, a low-income country (recently upgraded to a lower-middle-income country [6]) in South Asia, is a labour exporting country. Due to the lack of employment opportunities in the country, people are forced to go abroad for work [7]."*

(Page: 4, Line: 66-68)

*The majority of Nepalese migrant workers leave their children in Nepal when they go abroad. The children are looked after by the remaining family members in Nepal, mostly their grandparents as joint families are still common in Nepal.*

(Page: 5, Line: 91)

This study was a school-based, cross-sectional study conducted in two districts of Western Nepal, Baglung and Gulmi. *These are basically rural districts.*

(Page: 6; Line: 96-97)

Two municipalities, one from each district, with the high proportion of absent households (Galkot Municipality from Baglung and Musikot Municipality from Gulmi) were selected as the study sites. *These two areas were declared municipalities by the government in March 2017, after merging several Village Development Committees.*
