# Supplementary material for: Parental migration and psychological well-being of left-behind adolescents in Western Nepal
Source: PLoS One. 2021 Jan 28;16(1):e0245873. doi: 10.1371/journal.pone.0245873 (PMC7842897; doi:10.1371/journal.pone.0245873)
Supplement: Attachment — Submitted filename: Response to Reviewers.docx [file pone.0245873.s006.docx]

**Response to comments from the Editor:**

I ask if you can make one final change: in response to one of the reviewer comments, you removed what was previously Table 3 (simple linear regression). In the previous version, it was not clear that Table 3 was indeed a simple regression as it appeared that other factors were being controlled for. However, given your response and clarification, I feel it is important to include the simple regression as well as the multiple regression results. I would be very grateful if you could re-instate the deleted Table 3.

Thank you for your suggestion. We have re-instated Table 3 (simple linear regression) in the revised submission, and we have slightly modified the text to make it clearer.

Page: 14 Line: 215-217

"*Table 3 shows the results of simple linear regression analysis for the association of each independent variable with psychological well-being of adolescents, without controlling for other independent variables.*"

Page: 15 Line: 225-227

"*Table 4 presents the results of multiple linear regression analysis for the association of each independent variable with psychological well-being of adolescents, while controlling for other independent variables.*"
